# The Chemogenetic Receptor Ligand Clozapine N-Oxide Induces *in vivo* Neuroreceptor Occupancy and Reduces Striatal Glutamate Levels

**DOI:** 10.3389/fnins.2019.00187

**Published:** 2019-04-03

**Authors:** Simone Bærentzen, Agata Casado-Sainz, Denise Lange, Vladimir Shalgunov, Isabel Martinez Tejada, Mengfei Xiong, Elina T. L’Estrade, Fraser G. Edgar, Hedok Lee, Matthias M. Herth, Mikael Palner

**Affiliations:** ^1^Neurobiology Research Unit, Copenhagen University Hospital, Copenhagen, Denmark; ^2^Center for Translational Neuromedicine, University of Copenhagen, Copenhagen, Denmark; ^3^Institute of Aerospace Medicine, German Aerospace Center (DLR), Cologne, Germany; ^4^Department of Drug Design and Pharmacology, University of Copenhagen, Copenhagen, Denmark; ^5^Radiation Physics, Nuclear Medicine Physics Unit, Skånes University Hospital, Lund, Sweden; ^6^Department of Anesthesiology and Pediatric Anesthesiology, Yale University, New Haven, CT, United States; ^7^Department of Clinical Physiology, Nuclear Medicine and PET, Copenhagen University Hospital, Copenhagen, Denmark

**Keywords:** DREADD, CNO, clozapine, dopamine receptors, serotonin receptors, glutamate

## Abstract

Chemogenetic studies with the ligand clozapine N-oxide (CNO) are predicated upon the assumption that CNO is devoid of actions at natural neuroreceptors. However, recent evidence shows that CNO may be converted back to clozapine (CLZ) *in vivo*, which could yield plasma concentrations that may be sufficient to occupy inter alia dopamine D_2/3_ and serotonin 5HT_2A_ receptors in living brain. To test this phenomenon, we measured striatal dopamine D_2/3_ receptor occupancy with [^18^F]fallypride PET and serotonin 5HT_2A_ occupancy *ex vivo* using [^18^F]MH.MZ. We found a CNO dose-dependent effect on the availability of both neuroreceptor sites. In parallel MR spectroscopy experiments, we found that CNO reduced creatine + phosphcreatine (Cr+PCr) and increased *N*-acetylaspartate + *N*-acetylaspartylglutamate (NAA+NAAG) signals in the prefrontal cortex, and also reduced the glutamate signal in dorsal striatum, with peak effect at 2 mg/kg. Thus, our findings suggest that conversion of CNO to CLZ in living rats imparts significant occupancy at endogenous neuroreceptors and significant changes to neurometabolite levels.

## Introduction

Designer Receptors Exclusively Activated by Designer Drugs (DREADDs) are bioengineered from the muscarinic G protein-coupled receptor and have no natural ligand, but possess high agonistic affinity for the exogenous ligand clozapine N-oxide (CNO) (Armbruster et al., 2007). These receptors can be expressed with coupling to the Gq, Gi, and Gs signal transduction pathways, thus affording a powerful tool to study pathway-specific G-protein function of brain and peripheral nerves. CNO is a major metabolite of clozapine (CLZ) (Baldessarini et al., 1993), which is a widely used atypical antipsychotic medication with binding to a broad range of neuroreceptors, namely dopamine D_4_, D_2_, D_3_, serotonin 5-HT_2A_, 5-HT_2C_, muscarinic M_1_, M_2_, M_3_, M_4_, adrenergic α_1_ and α_2,_ as well as histamine H_1_ receptors. Furthermore, CLZ is also a high affinity agonist ligand for the DREADDs (Schotte et al., 1993; Bymaster et al., 1996; Zhang and Bymaster, 1999; Chen et al., 2015). While CNO is considered devoid of any specific binding other than to DREADD, recent studies suggest that CNO can convert back to CLZ in living rats (MacLaren et al., 2016) and dogs (Meier, 1975). Although CLZ was not detectable in plasma after a low administered dose of CNO (1 mg/kg) (Jann et al., 1993), a high dose of CNO (20 mg/kg) evoked measurable formation of plasma CLZ (Lin et al., 1996). CLZ has high uptake in rat brain, with a brain-to-plasma ratio of 24:1 (Baldessarini et al., 1993), predicting that much of the CLZ produced from CNO metabolism will accumulate in brain. In contrast, untransformed CNO has low permeability to the rat blood brain barrier (Gomez et al., 2017) and is therefore mainly confined to the plasma compartment of CLZ-treated rats (Baldessarini et al., 1993). Given this background, it has been suggested that the action of CNO on DREADDs in the brain is obtained solely via its metabolite CLZ (Gomez et al., 2017).

If CLZ is indeed the *in vivo* DREADD activator, it becomes imperative to confirm that a usual dose of CNO (or CLZ) does not interfere with other binding sites. CLZ is known to have *in vivo* occupancy at brain dopamine D_2/3_ receptors (Mukherjee et al., 2001; Olsen et al., 2008) as well as serotonin 5-HT_2A_ receptors in direct proportion to plasma concentrations (Knauer et al., 2008). Furthermore, CLZ treatment has been reported to alter *in vivo* levels of glutamate (Glu), *N*-acetylaspartate (NAA) and *N*-acetylaspartylglutamate (NAAG), albeit without direct relationship to the plasma CLZ concentration (Lidsky et al., 1993; Yamamoto and Cooperman, 1994; Arun et al., 2008; Krivoy et al., 2017). Therefore, brain concentrations of these neurochemcials can serve as surrogate indicators of central actions of the CNO metabolite.

To test our hypothesis that metabolism of CNO to CLZ *in vivo* induces blockade of D_2/3_ and 5-HT_2A_ receptors, we examined the CNO-induced *in vivo* receptor occupancy in conjunction with two available PET tracers, the dopamine D_2/3_ radioligand [^18^F]fallypride and the serotonin 5-HT_2A_ receptor radioligand [^18^F]MH.MZ. We chose to inject CNO 20 min before experimental procedures to ensure translatability with previously published animal studies (Agulhon et al., 2013; Urban et al., 2015; Baslow et al., 2016). Furthermore, to test for effects of the treatment on neurometabolites, we also examined neurochemical changes in the prefrontal cortex and striatum using Magnetic Resonance Spectroscopy (MRS). These experiments provide relevant information about dose-dependent effects of CNO at neuroreceptors in Long-Evans rats and may call for re-interpretation of some DREADD experiments published prior to the identification of CLZ as the *in vivo* activator after CNO administration.

## Materials and Methods

### Animals

Long-Evans rats were bred at the Department of Experimental Medicine Biocenter (AEM, University of Copenhagen). The rats were housed in groups of 2–4 animals per cage in a climate-controlled rodent facility with 12 h/12 h light/dark cycle. The rats were fed *ad libitum* and had free access to water. Female rats were used for the PET and MRS scans, while both male and female rats where used in the *ex vivo* experiment. All procedures were conducted in accordance with the FELASA guidelines for animal research and with approval from The Danish Animal Experiments Inspectorate (license number: 2016-15-0201-01031) as well as the Department of Experimental Medicine, University of Copenhagen.

### Chemicals

Radiochemicals were produced in house as described below. CNO was provided by BioNordika Denmark and all other chemicals were from standard vendors.

### Radiosyntheses of [^18^F]Fallypride

[^18^F]Fallypride was synthesized as previously described (Piel et al., 2014). In short, the tosyl-fallypride precursor (0.2 mg) in 1 mL dimethylsulfoxide (DMSO) was added to the dried [^18^F]KF-K_222_ complex via automated injection and was heated to 150°C for 20 min. The product was then purified using a semi-preparative HPLC method (Luna, 10 μm, C-18 column; 20% ethanol in 0.1% phosphoric acid in water-retention time 10 min). Purity of the isolated product was confirmed by analytical HPLC (Kinetic, C-18 with pre-column; 25% acetonitrile in 0.1% phosphoric acid in water; 1.5 mL/min – retention time 1.12 min). The radiochemical purity of [^18^F]fallypride was 99% and molar activity was 68 GBq/μmol. The resulting ethanol tracer solutions were diluted to the necessary volume in 0.1 M sterile phosphate buffer.

### Radiosyntheses of [^18^F]MH.MZ

[^18^F]MH.MZ was synthesized as previously described (Herth et al., 2008). In brief, MDL 105,725 was alkylated with 2-[^18^F]fluoroethyl tosylate ([^18^F]FEtTos) using an automated synthesis module. Identity of [^18^F]MH.MZ was confirmed by co-elution with non-radioactive [^19^F]MH.MZ standard on analytical HPLC (Luna 5 μm, C-18 column, acetonitrile/water/TFA 40/60/0.1 (v/v) – retention time 9.5 min). The radiochemical purity of [^18^F]MH.MZ was > 97% and molar activity was 22 GBq/μmol. The resulting ethanol tracer solutions were diluted to the necessary volume in 0.1 M sterile phosphate buffer.

### Dopamine D_2_ Receptor Occupancy

Five groups of female rats first received s.c. injections of 5% DMSO/saline vehicle (*n* = 16) or CNO [0.5 (*n* = 8), 2 (*n* = 6), 5 (*n* = 6) or 8 (*n* = 6) mg/kg] in 5% DMSO/saline followed 20 min later by an i.v. injection of approximately 10 MBq [^18^F]fallypride. The rats were anesthetized 40 min later with isoflurane (2–2.5% in oxygen) and placed in a homemade 2 × 2 rat insert in the aperture of a Siemens HRRT (High Resolution Research Tomograph) scanner (Keller et al., 2016) for a 45-min dynamic emission scan followed by a point source transmission scan (Tantawy et al., 2009, 2011). Scatter and attenuation corrections were performed, and the reconstructed PET image data were first cropped manually to a head-only image in PMOD v3.7, followed by motion correction with the PFUSEIT tool and summation for the purposes of registration. The images were corrected for body weight and injected dose using in-house code in MATLAB R2013a. The automatic co-registration with a tracer-specific PET template as target was executed in FSL 5.0.11 with the *flirt* command using the normalized correlation cost function with 12 degrees of freedom. A modified Logan reference tissue model with the cerebellum as reference region was used to calculate the binding potential (BP_ND_) in volumes of interest (VOIs) of bilateral dorsal or ventral striatum, as well as prefrontal cortex (Tantawy et al., 2009). Percent occupancy was calculated as the percent of the BP_ND_ or specific binding ratio at a given dose of CNO in relation to vehicle administration.

### Serotonin 5-HT_2A_ Receptor Occupancy

Five groups of male (m, round) and female (f, triangle) rats first received s.c. injections of 5% DMSO/saline vehicle (*n* = 7, 4m/3f) or CNO (0.5 (*n* = 8, 5m/3f), 2 (*n* = 8, 6m/2f), 5 (*n* = 7, 4m/3f) or 8 (*n* = 5, 5 m) mg/kg) in 5% DMSO/saline followed 20 min later by an i.v. injection of 200 **μ**L [^18^F]MH.MZ, approximately 10 MBq at the time of first injection. [^18^F]MH.MZ-injected rats were killed by decapitation at 60 min of tracer circulation, and the extracted brain was dissected into weighed frontal cortex and cerebellum samples. The concentration of tracer was measured in a Packard Cobra gamma counter, calculated per gram of tissue, and reported as specific binding ratio ((frontal cortex – cerebellum)/cerebellum). Percent occupancy was calculated as the percent of the BP_ND_ or specific binding ratio at a given dose of CNO in relation to vehicle administration.

### Glutamate Levels

Female rats (not the same rats as above) were anesthetized with isoflurane in oxygen, with induction at 3% and maintenance at 1.25–2.0% isoflurane during scans. All rats were scanned in the prefrontal cortex (30 min after) and in the right dorsal striatum (60 min after) after receiving s.c. injections of 5% DMSO/saline vehicle (*n* = 11/12) or CNO (0.5 (*n* = 4), 2 (*n* = 8), or 5 (*n* = 4) mg/kg) in 5% DMSO/saline. Rats were scanned at baseline and after treatment (excepting four of the 2 mg/kg CNO treated animals, which were only scanned after treatment, and one scan that failed in prefrontal cortex). Structural images were obtained with TurboRARE sequences. Local magnetic field homogeneity was adjusted in a 2 × 3 × 3 mm/18 μL VOI in the prefrontal cortex and in a 3 × 3 × 3 mm/27 μL VOI in the right dorsal striatum using FASTMAP. The MRS/1H-NMR spectrum was obtained using a STEAM (Stimulated Echo Acquisition Mode) sequence (TE: 3 ms, TR: 4000 ms, 400 ave, 4096 data points) with outer volume and VAPOR water suppression. All spectroscopy scans were performed on a Bruker BioSpec 94/30 USR MRI system (9.4 T, 30 cm bore, Bruker, Ettlingen, Germany) using a quadrature 86 mm rat body volume transmitter coil and 4 channel phase array receiver coil (Bruker, Ettlingen, Germany) with Paravision 5.3 software. MRS data was quantified with LCModel (Provencher, 1993) using simulated baseline and macro-molecule baseline.

### Statistics

All data are expressed as mean ± SD as calculated in Graphpad Prism v7.03. Significance of receptor occupancy changes was calculated with a one-way ANOVA with Dunnet’s multiple corrections test; ANOVA summary is attached in the Supplementary Material. Significance in neurometabolite levels to MRS was calculated using a two-way ANOVA with Sidak’s multiple corrections test. Significant levels are as follows: ^∗^*p* < 0.05, ^∗∗^*p* < 0.01 and ^∗∗∗^*p* < 0.001.

## Results

### Clozapine N-Oxide Induce Neuroreceptor Occupancy

**FIGURE 1:**
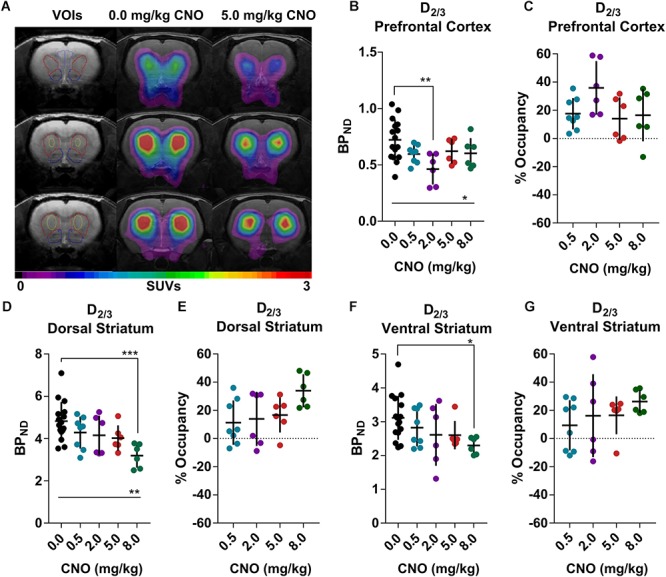
[^18^F]Fallypride binding in the rat prefrontal cortex and striatal regions **(A)**, 5 mg/kg Clozapine N-oxide (CNO) induced a noticeable decline in receptor availability in the same rat **(A)**. CNO induced occupancy on a group level at the dopamine D_2/3_ receptors in the prefrontal cortex **(B,C)** as well as dorsal **(D,E)** and ventral **(F,G)** striatum. Horizontal lines and ^∗^represents a significant effect of treatments, as measured with a one-way ANOVA (^∗^*p* < 0.05, ^∗∗^*p* < 0.01 and ^∗∗∗^*p* < 0.001).

**FIGURE 2:**
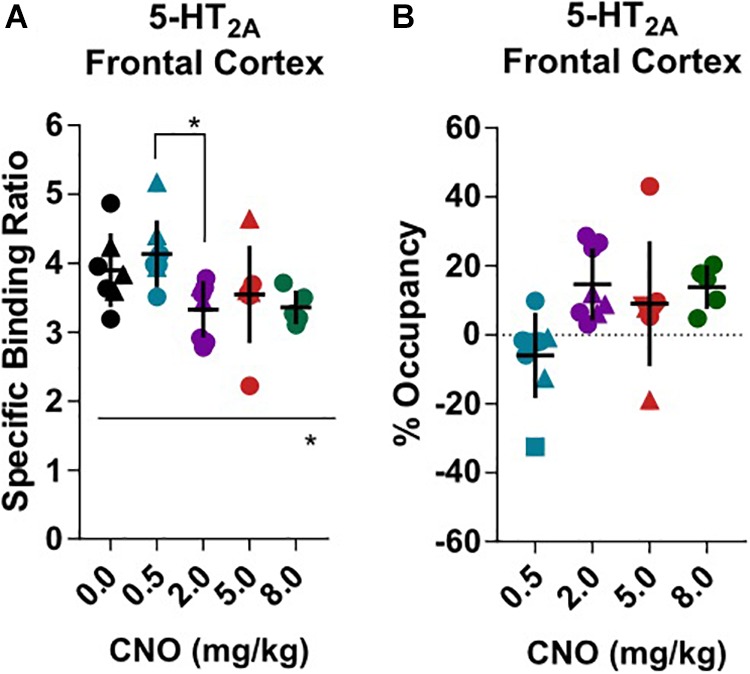
Serotonin 5HT_2A_ *ex vivo* specific binding ration of [^18^F]MH.MZ and the CNO induced occupancy. Male rats are round and females are triangular. Horizontal line and ^∗^represents a significant effect of treatments, as measured with a one-way ANOVA (^∗^*p* < 0.05, ^∗∗^*p* < 0.01 and ^∗∗∗^*p* < 0.001).

[^18^F]Fallypride, which binds with high affinity (*K*_D_: 0.03 nM) to dopamine D_2/3_ receptors *in vitro* (Mukherjee et al., 1995), is extensively used to measure dopamine or drug occupancy in dopamine D_2/3_ receptor rich regions of the living brain. After vehicle treatment, [^18^F]fallypride BP_ND_ was 0.72 ± 0.18 in the prefrontal cortex, 4.83 ± 0.91 in the dorsal striatum and 3.11 ± 0.65 in the ventral striatum (Figure 1). Pretreatment with 0.5 mg/kg CNO did not change the [^18^F]fallypride BP_ND_ in any region, but 2 mg/kg reduced BP_ND_ by 36% in the prefrontal cortex (BP_ND_ at 2 mg/kg CNO: 0.46 ± 0.14, Figure 1B,C). The effects of CNO 2 mg/kg in the prefrontal cortex were not evident at 5 and 8 mg/kg CNO. 5 mg/kg did not significantly affect the BP_ND_ in any region, whereas 8 mg/kg CNO reduced [^18^F]fallypride BP_ND_ by 34% in the dorsal and by 26% in ventral striatum (BP_ND_ at 8 mg/kg CNO: 3.19 ± 0.57, Figure 1D,E and 2.29 ± 0.25, respectively, Figure 1F,G). There was a significant difference of BP_ND_ between CNO treatments in the prefrontal cortex and dorsal striatum as calculated with an ordinary one-way ANOVA (Supplementary Tables 1–3). Furthermore, pretreatment with 0.5 mg/kg CNO did not significantly change the specific binding ratio of [^18^F]MH.MZ (a serotonin 5HT_2A_ receptor ligand), whereas pretreatment with 2, 5 and 8 mg/kg reduced the binding ratio by 15, 9, and 14%, respectively (Figure 2A,B). There was a significant effect of treatment between groups using an ordinary one-way ANOVA (Supplementary Table 4), although *post hoc* Dunnett’s test revealed that no single dose significantly altered the binding ratio relative to the vehicle group (Supplementary Table 4).

### Clozapine N-Oxide Reduces Striatal Glutamate Levels

**FIGURE 3:**
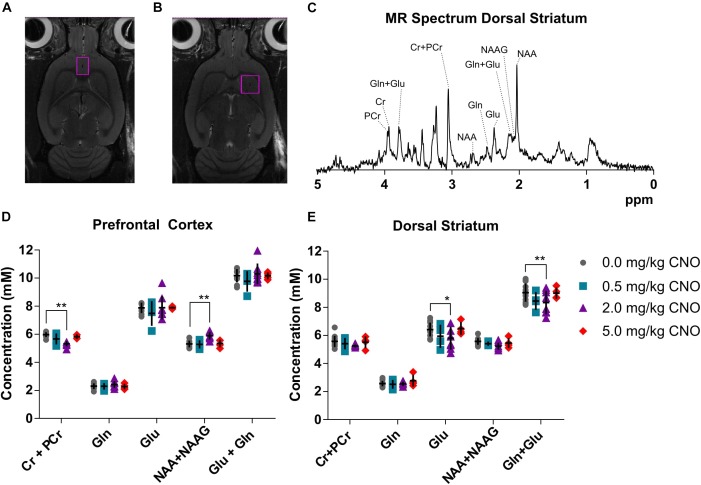
Neurometabolite concentrations as measured *in vivo* using MR spectroscopy. Cubic voxels 18 μL in the prefrontal cortex **(A)** and 27 μL dorsal striatum **(B)**. A sample spectrum **(C)** from the voxel in the dorsal striatum and metabolite concentrations in the voxels following pretreatments with different doses of CNO in the prefrontal cortex **(D)** and in the dorsal striatum **(E)**. ^∗^*p* < 0.05, ^∗∗^*p* < 0.01 and ^∗∗∗^*p* < 0.001 two-way ANOVA with Sidak’s multiple corrects test.

Baseline metabolite concentration of creatine + phosphocreatine (Cr+PCr), glutamine (Gln), glutamate (Glu), *N*-acetylaspartate + *N*-acetylaspartate glutamate (NAA + NAAG) and total glutamine + glutamate (Gln + Glu) were quantified from the obtained MR spectrum (Figure 3C) in prefrontal cortex (Figure 3D) and dorsal striatum (Figure 3E), as reported in detail in the Supplementary Material (Supplementary Table 5): GABA and glucose could not be reliably quantified in all rats using this voxel size and scan sequence. A representative spectrum and voxel placement are presented in Figure 3A–C. There was no effect of pretreatment with 0.5 mg/kg CNO in comparison to vehicle in either the prefrontal cortex or dorsal striatum. However, pretreatment with 2 mg/kg CNO induced a significant decrease in Cr+PCr (–11%, 5.32 ± 0.19 mM, *p* = 0.0032) and an increase in NAA+NAAG (14%, 5.89 ± 0.27 mM, *p* = 0.0095) concentrations in the prefrontal cortex. Furthermore, pretreatment with 2 mg/kg CNO also induced decreased glutamate (–9%, 5.79 ± 0.82 mM, *p* = 0.0251) and total glutamate + glutamine (–7%, 8.34 ± 0.84 mM, *p* = 0.0080) concentrations in the dorsal striatum (Statistics are reported in Supplementary Tables 7, 8).

## Discussion

The results confirm our hypothesis that CNO induces occupancy at dopamine D_2/3_ and serotonin 5-HT_2A_ receptors in Long-Evans rats, albeit without providing direct insight into the basis of this occupancy. It has been reported that CNO converts to CLZ *in vivo*, producing a peak CLZ plasma concentration between 10 and 30 min (Meier, 1975; MacLaren et al., 2016). Administration of CNO at a dose of 5 mg/kg generated 0.28 μM (about 92 ng/mL) CLZ in the plasma (MacLaren et al., 2016; Gomez et al., 2017) at 30 min after treatment. Baldessarini et al. found a similar CLZ concentration 1 h after an acute dose of 10 mg/kg CLZ i.p (Baldessarini et al., 1993). There is a two-phase correlation between plasma CLZ levels and the occupancy at dopamine D_2/3_ receptors (Mukherjee et al., 2001; Olsen et al., 2008); the plateau for the first phase occurred with doses in the range of 0.1 to 40 mg/kg CLZ i.p., These doses provoked a striatal occupancy of 20–50%, which is often considered as the therapeutic window for CLZ for the treatment of schizophrenia. In a rat [^18^F]fallypride PET study, Mukherjee et al. (2001) found a 30% occupancy level at 2 h after administration of 10 mg/kg CLZ. Although the exact occupancy by CLZ is dependent on the time of pretreatment and PET acquisition (Olsen et al., 2008), there is general agreement with our findings for CNO, which induced 26–39% dopamine D_2/3_ occupancy in the striatal regions at 1 h after injection of 8 mg/kg CNO. CLZ is known to induce dopamine release in the medial prefrontal cortex (Rowley et al., 2000) to a higher extent than in the dorsal striatum (Karoum and Egan, 1992). Therefore, it is likely that the inverted u-shape of CLZ-induced occupancy in the prefrontal cortex arises from dopamine release.

As mentioned above, the affinity of CNO for 5-HT_2A_ receptors (ID_50_ 1–2 mg/kg) exceeds that for dopamine D_2/3_ receptors (ID_50_ 20 mg/kg) (Schotte et al., 1993; Zhang and Bymaster, 1999). Present results support this by showing cortical 5-HT_2A_ receptor occupancy at low doses (Figure 2A,B) compared with D_2/3_ in the striatal regions. Knauer et al. found a CLZ dose-dependent occupancy of 5-HT_2A_ receptors, with a plasma concentration of 92 ng/mL giving 50–75% occupancy (Knauer et al., 2008). However, several questions arise from these [^18^F]MH.MZ experiments. First, CLZ occupancy of [^18^F]MH.MZ binding sites has not hitherto been established, so there is no direct literature we can compare to. Second, we do not know if CLZ and [^18^F]MH.MZ share the same binding site on the 5HT_2A_ receptors. We choose to include this experimental data because it provides some explanation as to why we detected effects on neurochemicals and D_2/3_ receptor occupancy at a CNO dose of 2 mg/kg. As previously noted, CLZ also bind to other neuroreceptors with higher affinity than toward dopamine D_2_ receptors. For the present, we can only assume that occupancy of these receptors will likely exceed that of the D_2_ receptors.

*In vivo* evidence of acute CLZ effects on neurometabolite levels is sparsely documented, although we know that chronic clozapine treatment does effect levels of glutamate, glutamine, creatine, NAA+NAAG and other metabolites (McLoughlin et al., 2009). Likewise, CLZ is without effect on the activity of nGCP II, the enzyme that catabolizes NAAG to glutamate (Flores and Coyle, 2003), or creatine kinase, the enzyme that catabolizes creatine to phosphocreatine (Assis et al., 2007; Scaini et al., 2013). The effects of CLZ on glutamate levels and glutamate signaling are particularly ambiguous. Some authors have reported that acute CLZ inhibited corticostriatal signaling and lowered glutamate release in PFC terminals (Lidsky et al., 1993; Yang and Wang 2005), while others have reported increased glutamate release in the PFC (Tanahashi et al., 2012). Interestingly, selective 5-HT_2A_ antagonists decrease glutamate concentrations in the dorsal striatum (Ansah et al., 2011), likely by blockade of the 5-HT_2A_ receptors, which are mainly located on the somatic and dendritic region of corticostriatal pyramidal neurons (Miner et al., 2003). We suppose that 5-HT_2A_ inhibition of glutamate release in dorsal striatum could precede the glutamate release potentiated by other means, as the inhibitory effect on glutamate was evident with a CNO dose (2 mg/kg) lower than that causing discernible occupancy at D_2/3_ receptors (5 mg/kg). Such a bidirectional activation and inhibition could explain the effects that we and others observe, but further studies are needed to elucidate the neurochemical basis of the observed effects of CNO and to confirm that these effects are indeed precipitated by conversion of CNO to CLZ in the living rodent. However, these data confirm that CNO is not a neurochemically inert drug and call for caution in the attribution of effects of CNO in the context of chemogenetic (DREADD) ligand studies.

## Data Availability

Raw data can be obtained by contacting the corresponding author.

## Author Contributions

SB carried out most animal experiments, analyzed the data and assisted in writing the manuscript. AC-S assisted in the animal experiments, data analysis and writing of the manuscript. DL automated PET image co-registration, motion correction and kinetic modeling. VS synthesized [^18^F]MH.MZ and assisted in PET experiments. IT assisted in PET data analysis. MX assisted in the animal experiments. EL established the synthesis and novel HPLC method of [^18^F]fallypride, and assisted in PET experiments and data analysis. FE synthesized [^18^F]fallypride and assisted in PET experiments. HL established the MRS sequence, assisted in analyzing the MRS spectra, and edited the manuscript. MMH planned the radiochemical and PET experiments, discussed the data, and edited the manuscript. MP planned the whole study, established the PET imaging protocol, carried out experiments, analyzed PET and MRS data, and wrote the manuscript. All authors have read and approved the manuscript.

## Conflict of Interest Statement

The authors declare that the research was conducted in the absence of any commercial or financial relationships that could be construed as a potential conflict of interest.
