# Supplementary material for: The Chemogenetic Receptor Ligand Clozapine N-Oxide Induces in vivo Neuroreceptor Occupancy and Reduces Striatal Glutamate Levels
Source: Front Neurosci. 2019 Apr 3;13:187. doi: 10.3389/fnins.2019.00187 (PMC6456655; doi:10.3389/fnins.2019.00187)
Supplement: Supplementary file 1 [file Table_1.docx]

Supplementary Material

The chemogenetic receptor ligand clozapine N-oxide induces *in vivo* neuroreceptor occupancy and reduces striatal glutamate levels.

**Simone Bærentzen^1,2^, Agata Casado-Sainz^1^, Denise Lange^1,3^, Vladimir Shalgunov^3^, Isabel Martinez Tejada^1^, Mengfei Xiong^1,4^, Elina T. L’Estrade^1,3,4^, Fraser G. Edgar^3^, Hedok Lee^5^, Matthias M. Herth^1,3,6^, and Mikael Palner^1,2^***

^1^Neurobiology Research Unit, Copenhagen University Hospital, Copenhagen, Denmark.

^2^Center for Translational Neuromedicine, University of Copenhagen, Copenhagen, Denmark.

^3^ Institute of Aerospace Medicine, German Aerospace Center (DLR), Cologne, Germany

^4^Department of Drug Design and Pharmacology, University of Copenhagen, Copenhagen, Denmark.

^5^ Radiation Physics, Nuclear Medicine Physics Unit, Skånes University Hospital, Lund, Sweden.

^6^ Department of Anesthesiology and Pediatric Anesthesiology, Yale University, New Haven, CT USA.

^7^ Department of Clinical Physiology, Nuclear Medicine and PET, Copenhagen University Hospital, Copenhagen, Denmark.

Corresponding Author
mikael.palner@nru.dk

# Supplementary Data

## Dopamine D_2_ receptor occupancy

Statistical results from one-way ANOVA with Dunnett’s Multiple Comparisons Test

| Supplementary table 1 - Dorsal Striatum | | |  |  | |  |  |
| --- | --- | --- | --- | --- | --- | --- | --- |
| One-way ANOVA | SS | DF | F (DFn, DF,d) | | | Significant? | P Value |
| Treatment (Between Columns) | 12.41 | 4 | F (4, 37) = 4.752 | | | ** | 0.0034 |
| Residual (Within Columns) | 24.16 | 37 |  | | |  |  |
|  |  |  |  |  |  |  |  |
| Dunnett’s Multiple Comparisons Test | Mean Diff | SE on Diff | n1 | n2 | DF | Significant? | P Value |
| 0.0 vs. 0.5 | 0.5443 | 0.3499 | 16 | 8 | 37 | ns | 0.4227 |
| 0.0 vs. 2.0 | 0.6724 | 0.3868 | 16 | 6 | 37 | ns | 0.3156 |
| 0.0 vs. 5.0 | 0.805 | 0.3868 | 16 | 6 | 37 | ns | 0.1661 |
| 0.0 vs. 8.0 | 1.637 | 0.3868 | 16 | 6 | 37 | *** | 0.0006 |

| Supplementary table 2 - Ventral Striatum | | |  |  |  | |  |  |
| --- | --- | --- | --- | --- | --- | --- | --- | --- |
| One-way ANOVA | SS | DF | | F (DFn, DF,d) | | | Significant? | P Value |
| Treatment (Between Columns) | 3.548 | 4 | | F (4, 37) = 2.356 | | | ns | 0.0714 |
| Residual (Within Columns) | 13.93 | 37 | |  | | |  |  |
|  |  |  | |  |  |  |  |  |
| Dunnett’s Multiple Comparisons Test | Mean Diff | SE on Diff | | n1 | n2 | DF | Significant? | P Value |
| 0.0 vs. 0.5 | 2.822 | 0.2914 | | 0.2657 | 16 | 37 | ns | 0.7310 |
| 0.0 vs. 2.0 | 2.61 | 0.5035 | | 0.2937 | 16 | 37 | ns | 0.3287 |
| 0.0 vs. 5.0 | 2.601 | 0.5123 | | 0.2937 | 16 | 37 | ns | 0.3126 |
| 0.0 vs. 8.0 | 2.297 | 0.8166 | | 0.2937 | 16 | 37 | * | 0.0335 |

| Supplementary table 3 - Prefrontal Cortex | | |  |  |  | |  |  |
| --- | --- | --- | --- | --- | --- | --- | --- | --- |
| One-way ANOVA | SS | DF | | F (DFn, DF,d) | | | Significant? | P Value |
| Treatment (Between Columns) | 0.3175 | 4 | | F (4, 37) = 3.803 | | | * | 0.0109 |
| Residual (Within Columns) | 0.7722 | 37 | |  | | |  |  |
|  |  |  | |  |  |  |  |  |
| Dunnett’s Multiple Comparisons Test | Mean Diff | SE on Diff | | n1 | n2 | DF | Significant? | P Value |
| 0.0 vs. 0.5 | 0.1268 | 0.06256 | | 16 | 8 | 37 | ns | 0.1525 |
| 0.0 vs. 2.0 | 0.2591 | 0.06916 | | 16 | 6 | 37 | ** | 0.0023 |
| 0.0 vs. 5.0 | 0.1011 | 0.06916 | | 16 | 6 | 37 | ns | 0.4043 |
| 0.0 vs. 8.0 | 0.1193 | 0.06916 | | 16 | 6 | 37 | ns | 0.2657 |

## Serotonin 5HT_2A_ receptor occupancy

| Supplementary table 4 - Frontal Cortex | | |  |  |  | |  |  |
| --- | --- | --- | --- | --- | --- | --- | --- | --- |
| One-way ANOVA | SS | DF | | F (DFn, DF,d) | | | Significant? | P Value |
| Treatment (Between Columns) | 3.596 | 4 | | F (4, 30) = 3.479 | | | * | 0.0189 |
| Residual (Within Columns) | 7.753 | 30 | |  | | |  |  |
|  |  |  | |  |  |  |  |  |
| Dunnett’s Multiple Comparisons Test | Mean Diff | SE on Diff | | n1 | n2 | DF | Significant? | P Value |
| 0.0 vs. 0.5 | -0.2333 | 0.2631 | | 7 | 8 | 30 | ns | 0.9657 |
| 0.0 vs. 2.0 | 0.5694 | 0.2631 | | 7 | 8 | 30 | ns | 0.2405 |
| 0.0 vs. 5.0 | 0.3537 | 0.2717 | | 7 | 7 | 30 | ns | 0.7955 |
| 0.0 vs. 8.0 | 0.54 | 0.2977 | | 7 | 5 | 30 | ns | 0.4406 |
| 0.5 vs. 2.0 | 0.8027 | 0.2542 | | 8 | 8 | 30 | * | 0.0250 |
| 0.5 vs. 5.0 | 0.587 | 0.2631 | | 8 | 7 | 30 | ns | 0.2110 |
| 0.5 vs. 8.0 | 0.7733 | 0.2898 | | 8 | 5 | 30 | ns | 0.0822 |

## Clozapine N-oxide reduces striatal glutamate levels

| \| Supplementary table 5 – MR Spectroscopy Prefrontal Cortex \|  \| \| --- \| --- \| | | | | |
| --- | --- | --- | --- | --- | --- | --- |
|  | Vehicle | 0.5 mg/kg CNO | 2.0 mg/kg CNO | 5.0 mg/kg CNO |
| Cr+PCr | 5.98 ± 0.17 (11) | 5.67 ± 0.40 (4) | 5.32 ± 0.19 (8) | 5.83 ± 0.17 (4) |
| Gln | 2.30 ± 0.20 (11) | 2.29 ± 0,24 (4) | 2.41 ± 0.24 (8) | 2.27 ± 0.20 (4) |
| Glu | 7.88 ± 0.38 (11) | 7.48 ± 0.88 (4) | 7.89 ± 0.83 (8) | 7.89 ± 0.08 (4) |
| NAA+NAAG | 5.31 ± 0.24 (11) | 5.28 ± 0.30 (4) | 5.89 ± 0.27 (8) | 5.35 ± 0.28 (4) |
| Gln+Glu | 10.18 ± 0.46 (11) | 9.77 ± 0.74 (4) | 10.30 ± 0.73 (8) | 10.16 ± 0.25 (4) |

| Supplementary table 6 – MR Spectroscopy Prefrontal Cortex | | | |  | |  |  |
| --- | --- | --- | --- | --- | --- | --- | --- |
| Two-way ANOVA | SS | DF | F (DFn, DF,d) | | | Significant? | P Value |
| Interaction | 4.319 | 12 | F (12, 115) = 2.056 | | | * | 0.0253 |
| Metabolites | 750.3 | 4 | F (4, 115) = 1071 | | | **** | <0.0001 |
| Treatment | 1.028 | 3 | F (3, 115) = 1.956 | | | ns |  |
|  |  |  |  |  |  |  |  |
| Sidak’s Multiple Comparisons Test | Mean Diff | SE on Diff | n1 | n2 | DF | Significant? | P Value |
| Cr + PCr |  |  |  |  |  |  |  |
| 0.0 mg/kg CNO vs. 0.5 mg/kg CNO | 0.3034 | 0.2443 | 11 | 4 | 115 | ns | 0.5195 |
| 0.0 mg/kg CNO vs. 2.0 mg/kg CNO | 0.6504 | 0.1944 | 11 | 8 | 115 | ** | 0.0033 |
| 0.0 mg/kg CNO vs. 5.0 mg/kg CNO | 0.1457 | 0.2443 | 11 | 4 | 115 | ns | 0.9102 |
|  |  |  |  |  |  |  |  |
| Gln |  |  |  |  |  |  |  |
| 0.0 mg/kg CNO vs. 0.5 mg/kg CNO | 0.01214 | 0.2443 | 11 | 4 | 115 | ns | >0.9999 |
| 0.0 mg/kg CNO vs. 2.0 mg/kg CNO | -0.1179 | 0.1944 | 11 | 8 | 115 | ns | 0.9062 |
| 0.0 mg/kg CNO vs. 5.0 mg/kg CNO | 0.02464 | 0.2443 | 11 | 4 | 115 | ns | 0.9995 |
|  |  |  |  |  |  |  |  |
| Glu |  |  |  |  |  |  |  |
| 0.0 mg/kg CNO vs. 0.5 mg/kg CNO | 0.3973 | 0.2443 | 11 | 4 | 115 | ns | 0.2871 |
| 0.0 mg/kg CNO vs. 2.0 mg/kg CNO | -0.009 | 0.1944 | 11 | 8 | 115 | ns | >0.9999 |
| 0.0 mg/kg CNO vs. 5.0 mg/kg CNO | -0.01375 | 0.2443 | 11 | 4 | 115 | ns | >0.9999 |
|  |  |  |  |  |  |  |  |
| NAA+NAAG |  |  |  |  |  |  |  |
| 0.0 mg/kg CNO vs. 0.5 mg/kg CNO | 0.02764 | 0.2443 | 11 | 4 | 115 | ns | 0.9993 |
| 0.0 mg/kg CNO vs. 2.0 mg/kg CNO | -0.5816 | 0.1944 | 11 | 8 | 115 | * | 0.0102 |
| 0.0 mg/kg CNO vs. 5.0 mg/kg CNO | -0.03986 | 0.2443 | 11 | 4 | 115 | ns | 0.9978 |
|  |  |  |  |  |  |  |  |
| Glu + Gln |  |  |  |  |  |  |  |
| 0.0 mg/kg CNO vs. 0.5 mg/kg CNO | 0.4093 | 0.2443 | 11 | 4 | 115 | ns | 0.2626 |
| 0.0 mg/kg CNO vs. 2.0 mg/kg CNO | -0.1268 | 0.1944 | 11 | 8 | 115 | ns | 0.8863 |
| 0.0 mg/kg CNO vs. 5.0 mg/kg CNO | 0.01157 | 0.2443 | 11 | 4 | 115 | ns | >0.9999 |

| \| Supplementary table 7 – MR Spectroscopy Dorsal Striatum \|  \| \| --- \| --- \| | | | | |
| --- | --- | --- | --- | --- | --- | --- |
|  | Vehicle | 0.5 mg/kg CNO | 2.0 mg/kg CNO | 5.0 mg/kg CNO |
| Cr+PCr | 5.57 ± 0.40 (12) | 5.41 ± 0.40 (4) | 5.25 ± 0.10 (8) | 5.51 ± 0.41 (4) |
| Gln | 2.56 ± 0.19 (12) | 2.51 ± 0,31 (4) | 2.55 ± 0.16 (8) | 2.77 ± 0.43 (4) |
| Glu | 6.42 ± 0.49 (12) | 5.94 ± 0.83 (4) | 5.79 ± 0.82 (8) | 6.49 ± 0.46 (4) |
| NAA+NAAG | 5.58 ± 0.25 (12) | 5.41 ± 0.14 (4) | 5.23 ± 0.25 (8) | 5.45 ± 0.36 (4) |
| Gln+Glu | 9.04 ± 0.66 (12) | 8.44 ± 0.64 (4) | 8.34 ± 0.74 (8) | 9.01 ± 0.41 (4) |

| Supplementary table 8 – MR Spectroscopy Dorsal Striatum | | | |  | |  |  |
| --- | --- | --- | --- | --- | --- | --- | --- |
| Two-way ANOVA | SS | DF | F (DFn, DF,d) | | | Significant? | P Value |
| Interaction | 1.978 | 12 | F (12, 120) = 0.7246 | | | Ns |  |
| Metabolites | 430.3 | 4 | F (4, 120) = 472.7 | | | **** | <0.0001 |
| Treatment | 4.842 | 3 | F (3, 120) = 7.094 | | | *** | 0.0002 |
|  |  |  |  |  |  |  |  |
| Sidak’s Multiple Comparisons Test | Mean Diff | SE on Diff | n1 | n2 | DF | Significant? | P Value |
| Cr+PCr |  |  |  |  |  |  |  |
| 0.0 mg/kg CNO vs. 0.5 mg/kg CNO | 0.1591 | 0.2754 | 12 | 4 | 120 | ns | 0.9175 |
| 0.0 mg/kg CNO vs. 2.0 mg/kg CNO | 0.3195 | 0.2177 | 12 | 8 | 120 | ns | 0.3748 |
| 0.0 mg/kg CNO vs. 5.0 mg/kg CNO | 0.06133 | 0.2754 | 12 | 4 | 120 | ns | 0.9946 |
|  |  |  |  |  |  |  |  |
| Gln |  |  |  |  |  |  |  |
| 0.0 mg/kg CNO vs. 0.5 mg/kg CNO | 0.05217 | 0.2754 | 12 | 4 | 120 | ns | 0.9966 |
| 0.0 mg/kg CNO vs. 2.0 mg/kg CNO | 0.01842 | 0.2177 | 12 | 8 | 120 | ns | 0.9997 |
| 0.0 mg/kg CNO vs. 5.0 mg/kg CNO | -0.2028 | 0.2754 | 12 | 4 | 120 | ns | 0.8450 |
|  |  |  |  |  |  |  |  |
| Glu |  |  |  |  |  |  |  |
| 0.0 mg/kg CNO vs. 0.5 mg/kg CNO | 0.4822 | 0.2754 | 12 | 4 | 120 | ns | 0.2278 |
| 0.0 mg/kg CNO vs. 2.0 mg/kg CNO | 0.6238 | 0.2177 | 12 | 8 | 120 | * | 0.0147 |
| 0.0 mg/kg CNO vs. 5.0 mg/kg CNO | -0.07508 | 0.2754 | 12 | 4 | 120 | ns | 0.9901 |
|  |  |  |  |  |  |  |  |
| NAA+NAAG |  |  |  |  |  |  |  |
| 0.0 mg/kg CNO vs. 0.5 mg/kg CNO | 0.1671 | 0.2754 | 12 | 4 | 120 | ns | 0.9059 |
| 0.0 mg/kg CNO vs. 2.0 mg/kg CNO | 0.3496 | 0.2177 | 12 | 8 | 120 | ns | 0.2974 |
| 0.0 mg/kg CNO vs. 5.0 mg/kg CNO | 0.1273 | 0.2754 | 12 | 4 | 120 | ns | 0.9551 |
|  |  |  |  |  |  |  |  |
| Gln+Glu |  |  |  |  |  |  |  |
| 0.0 mg/kg CNO vs. 0.5 mg/kg CNO | 0.6005 | 0.2754 | 12 | 4 | 120 | ns | 0.0906 |
| 0.0 mg/kg CNO vs. 2.0 mg/kg CNO | 0.7083 | 0.2177 | 12 | 8 | 120 | ** | 0.0044 |
| 0.0 mg/kg CNO vs. 5.0 mg/kg CNO | 0.03775 | 0.2754 | 12 | 4 | 120 | ns | 0.9987 |
